# The telomere‐to‐telomere gap‐free genome of four rice parents reveals SV and PAV patterns in hybrid rice breeding

**DOI:** 10.1111/pbi.13880

**Published:** 2022-07-12

**Authors:** Yilin Zhang, Jun Fu, Kai Wang, Xue Han, Tianze Yan, Yanning Su, Yanfeng Li, Zechuan Lin, Peng Qin, Chenjian Fu, Xing Wang Deng, Degui Zhou, Yuanzhu Yang, Hang He

**Affiliations:** ^1^ School of Advanced Agriculture Sciences and School of Life Sciences, State Key Laboratory of Protein and Plant Gene Research Peking University Beijing China; ^2^ Peking University Institute of Advanced Agricultural Sciences Shandong Laboratory of Advanced Agricultural Sciences at Weifang Weifang China; ^3^ Key Laboratory of Southern Rice Innovation & Improvement, Ministry of Agriculture and Rural Affairs, Hunan Engineering Laboratory of Disease and Pest Resistant Rice Breeding Yuan Longping High‐Tech Agriculture Co., Ltd. Changsha China; ^4^ State Key Laboratory of Hybrid Rice Hunan Hybrid Rice Research Center Changsha China; ^5^ College of Plant Science & Technology Huazhong Agricultural University Wuhan China; ^6^ Guangdong Key Laboratory of New Technology in Rice Breeding Rice Research Institute, Guangdong Academy of Agricultural Sciences Guangzhou China; ^7^ State Key Laboratory for Conservation and Utilization of Subtropical Agro‐Bioresources, College of Life Sciences South China Agricultural University Guangzhou China

**Keywords:** hybrid‐rice, gap‐free genome, structure variation, heterosis

Xiangling628S (XL628S), Longke638S (LK638S), and Jing4155S (J4155S) are excellent two‐line sterile lines, and Huazhan (HZ) and Wu‐Shan‐Si‐Miao (WSSM) are both two‐line and three‐line restorers. LK638S and J4155S are derived from XL628S, belonging to the same pedigree, while WSSM is derived from HZ, belonging to another pedigree. Between 2018 and 2020, the planting area of JLYHZ, JLY534, and LLYHZ ranked among top 3 of China's hybrid rice (Figure 1a). In the published genomes, 82 and 129 gaps remained for J4155S and Huazhan assemblies, respectively, while no assemblies for LK638S and XL628S were observed (Qin *et al*., 2021; Zhang *et al*., 2021). To analyze the genomic basis of modern elite hybrid rice heterosis, high‐quality genome reference of core restorer lines and sterile lines is necessary.

**Figure 1:**
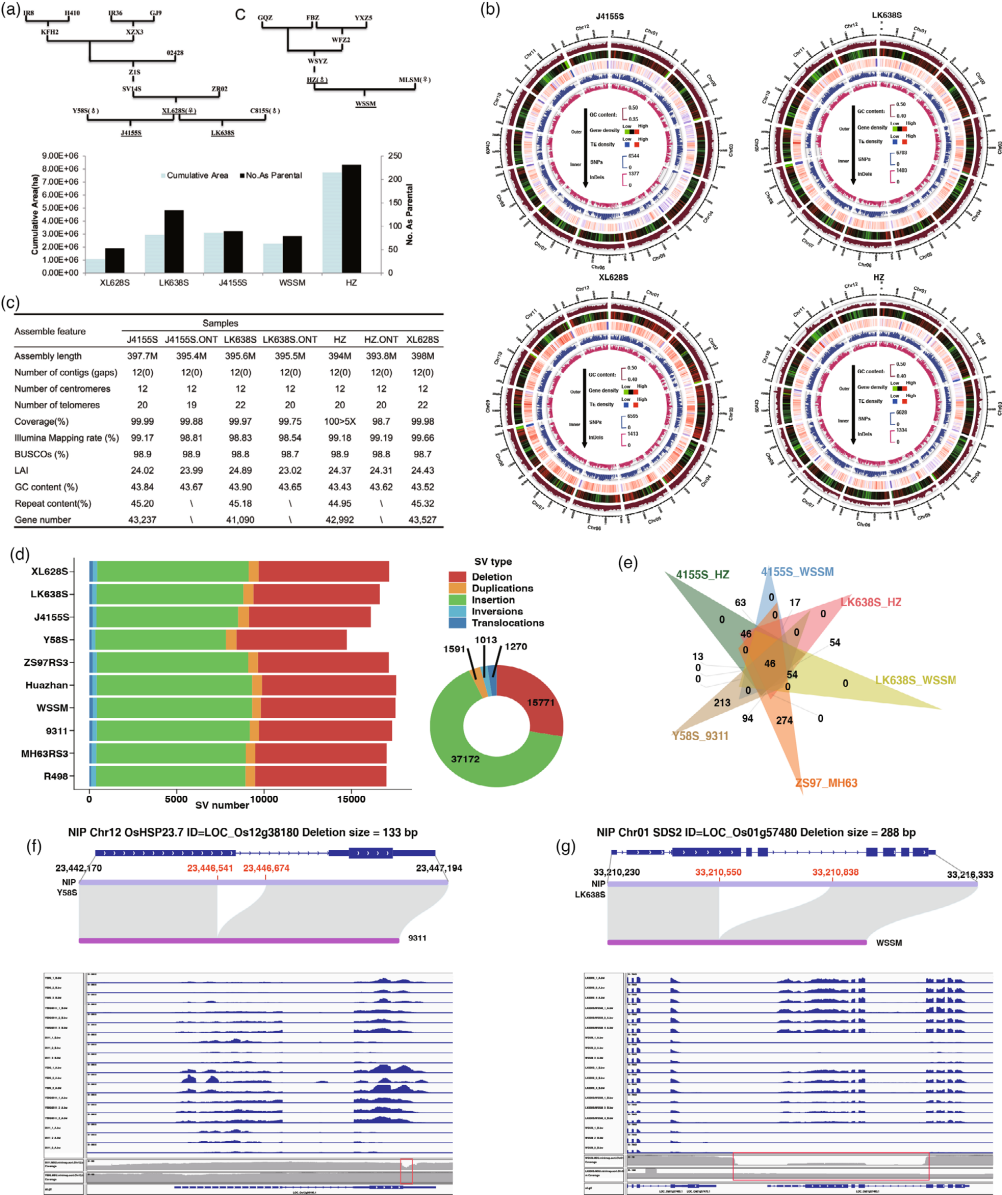
Overview of four genomes and PAV patterns in hybrid rice. (a) Four *indica* pedigree and agronomic traits. (b) Overview of the four rice genomes. (c) Statistics of the assembly features for four genomes. (d) Number of SVs between NIP and ten *indica* genomes. (e) Venn diagram of genes that six hybrid combinations of complementary utilization affected by PAV. (f) *OsHSP23.7* gene overview of SV and IGV. From top to bottom, the Y58S, F1, 9311 expression in the panicle (a) and leaf (b), Y58S and 9311 Pacbio genome data. (g) *SDS2* gene overview of SV and IGV. From top to bottom, the LK638S, F1, WSSM expression in *the* panicle (a) and leaf (b), LK638S and WSSM Pacbio genome data. The mapped reads showed different abundances in three genomes and SV in two parents genomes (f, g).

We adopted an assembly strategy that integrated Pacbio HiFi and Oxford Nanopore Technology (ONT) ultra‐long reads and then generated four gap‐free genomes. The estimated genome size was 400 Mb by K‐mer analysis. For HiFi, the assembly of contigs by hifiasm realized the gapless assembly by utilizing the gap‐filling method PGA (Li *et al*., 2021). For ONT, the ultra‐long reads directly assembled 12 gapless contigs by Nextdenovo which could be entirely anchored to chromosomes completely. Finally, the HiFi and short reads were combined to polish the ONT assembly. The Illumina mapping rates were from 98.83% to 99.66% and the coverage rates were more than 99.97%. The completeness of the genomes ranged from 98.7% to 98.9% by BUSCO, and LAI values were from 24.02 to 24.89. The centromeric regions and 20–22 telomeres were identified on each of genomes. These results demonstrate the high quality of genome assembly. The contents of the repeats were from 44.95% to 45.32%, while from 41 090 to 43 527, protein‐coding gene structures were predicted (Figure 1c). Using 500‐kb intervals, the GC content, gene, TE, SNP, and InDel density were counted in the genomes (Figure 1b).

To assess plant genome assembly quality using different strategies, we performed assemblies with HiFi, ONT, or combination strategy and compared the accuracy and completeness. BUSCO of HiFi was 0.1% higher than that of ONT assemblies (98.9% for HiFi and 98.8% for ONT), while mapping rates of HiFi were also 0.3% higher than those of ONT assemblies (99.17% for HiFi and 98.81% for ONT), indicating that HiFi assemblies had advantages in accuracy. The HiFi and ONT assemblies generated 12 centromeres and 19–22 telomeres, respectively (Figure 1c). Continuity of ONT assembly is higher than that of HiFi assembly (12 contigs for ONT and 786 contigs for HiFi draft assemblies). However, the combination strategy presented both high accuracy by HiFi assembly and continuity and correctly gap‐filling by ONT assembly, which was suggested for preference of future plant genome assembly.

To analyze how SV affects hybrid rice breeding, we selected six other high‐quality‐assembled *indica*, namely, MH63RS3, ZS97RS3, R498, Y58S, 9311 and WSSM, which were also wildly used as parents of hybrid rice breeding. Using the *Nipponbare* (NIP) as reference, we compared the SNPs, indels, and SV of the six reported and four *indica* genomes in this study. 2 210 644–2 731 354 SNPs and 422 858–52 6481 indels between the NIP and 10 *indica* genome were identified. A total of 56 817 non‐redundant SVs were cataloged, including 52 943 PAVs (Figure 1d). We noticed that fewer SVs were detected among sterile lines (16 108–17 139) than among restorer lines (17 014–17 560), suggesting large variations were more introduced from restorer lines (Figure 1d). In particular, fewer SVs were detected among two‐line sterile lines (14 734–16 621) than that among three‐line sterile lines (17 139), but the proportion of SVs derived from *japonica* in two‐line sterile lines was increased (33.6% in two‐line sterile lines and 30.9% in three‐line sterile lines). Of 10 *indica* genomes, an average of 1360 functional genes (Peng *et al*., 2019) were affected by PAVs. 565 genes that were influenced in all 10 *indica* may have differentiated between *indica* and *japonica*, including *OsLBD37*, *SaM*, *OsMFT1*, and *OsF3H*. Meanwhile, 299 genes were uniquely affected in one of the 10 genomes, including *Ghd8* (ZS97), *NF‐YC12* (9311), and *rhd1* (XL628S).

The 10 *indica* included the three‐line combination MH63/ZS97 and the two‐line combinations Y58S/93–11, J4155S/HZ, LK638S/HZ, J4155S/WSSM, and LK638S/WSSM, all among the widest promotion area from 2010 to 2020, present the three stages of the hybrid rice breeding. The complementary gene utilization was estimated by the PAVs that occurred in one parent but did not occur in another parent. According to analysis, among 593–738 complementary genes in the six combinations, and only 46 genes are utilized by all three types of combinations, and 213–274 genes are utilized (Figure 1e). The number of heterosis genes utilized individually exceeds the number of universally presenting key heterosis genes. The expression patterns of genes affected by PAV in F1 were further demonstrated by RNA‐seq. For two‐line including Y58S/9311 and LK638S/WSSM, 698 and 593 functional genes are complementary in the parental genome, respectively. Among these genes, 69 were additively, 143 were partially‐dominant, 258 were dominantly, and 338 were over‐dominantly expressed. For example, *OsHSP23.7* sequence of Y58S was the same as NIP, when it has a 133 bp deletion on the exon of 9311, and *OsHSP23.7* of F1 was dominantly expressed in panicle (A) and over‐dominantly expressed in leaf (B) (Figure 1f), suggesting that F1 enhances drought and salt tolerance introduced from Y58S (Zou, 2012). *SDS2* sequence of LK638S was same as NIP, when it has a 288 bp deletion on the exon of WSSM, and *SDS2* of F1 was dominantly expressed in the panicle and leaf (Figure 1g), suggesting the F1 rice blast resistance introduced from LK638S (Fan *et al*., 2018).

In this study, four parents of two‐line hybrid rice were *de novo* assembled on different platforms. The combination assembly strategy presented four high‐quality gap‐free assembled genomes. Using these four and the other six reported rice genomes, 52 943 PAVs were identified. For three‐line hybrids, 738 complementary utilized functional genes were identified, while 593–698 complementary utilized functional genes were identified for two‐line hybrids. Among them, only 46 genes were in common, suggesting distinct PAV genes in two‐line and three‐line hybrids. Furthermore, for Y58S/9311 and LK638S/WSSM, RNA‐seq demonstrated that 27.7% (194 of 698) and 26.1% (155 of 593) complementary utilized genes were over‐dominantly expressed in hybrids, respectively, suggesting SV plays an important role in gene expression heterosis. These four gap‐free rice genomes and SV complementary patterns in rice cultivars will contribute to future genomics research and breeding of excellent hybrid rice.

## Conflict of interest

The authors declare no competing interests.

## Authors' contributions

H.H. and Y.Y. designed the project. Y.Z., J.F., K.W., and X.H. performed the data analysis. Y.Z., J.F., and K.W. performed the experiments. Y.Z., H.H., D.Z, and X.W.D. wrote the paper. All authors read and approved the final manuscript.

## Data Availability

All the raw sequencing data and genome assembly generated for this project are archived at the National Genomics Data Center under BioProject accession no. PRJCA008812. All genome assemblies with annotations and structure variants VCF files are available at the GitHub (https://github.com/yilinZhang‐bio/Four‐rice‐gap‐free‐genome).
